# Supplementary material for: Molecular basis of the inositol deacylase PGAP1 involved in quality control of GPI-AP biogenesis
Source: Nat Commun. 2024 Jan 2;15:8. doi: 10.1038/s41467-023-44568-2 (PMC10761859; doi:10.1038/s41467-023-44568-2)
Supplement: Supplementary file 1 — Supplementary Information [file 41467_2023_44568_MOESM1_ESM.pdf]

Title: Molecular basis of the inositol deacylase PGAP1 involved in quality control of GPI-AP biogenesis

Authors list:

Jingjing Hong[1,†], Tingting Li[1,†], Yulin Chao[2,†], Yidan Xu[1], Zhini Zhu[2], Zixuan Zhou[2], Weijie Gu[1], Qianhui Qu[2,*], Dianfan Li[2,*]

Supplementary Information

Fig. S1-S13

Table S1-S2

Supplementary References 1-11

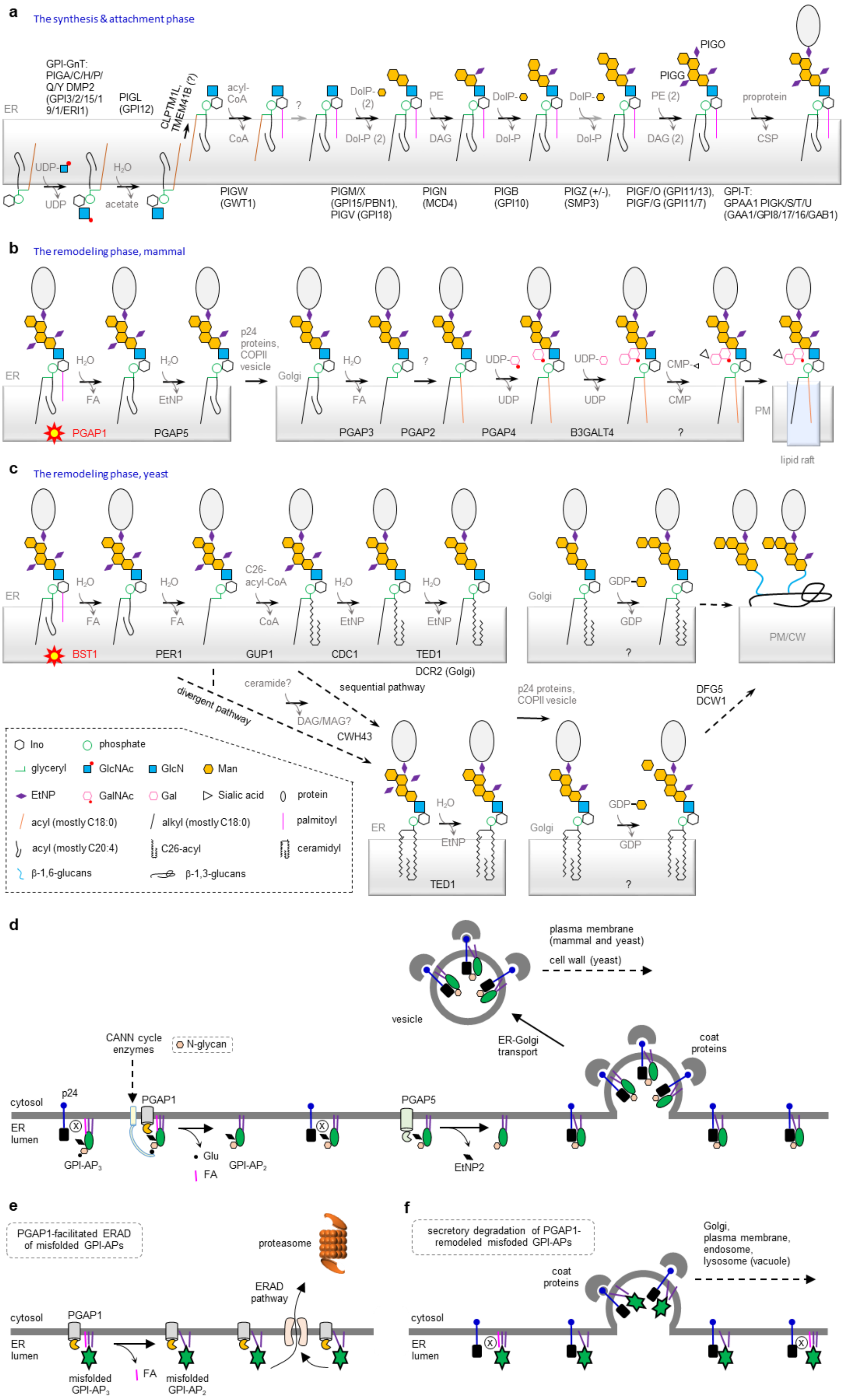

See next page for caption

Fig. S1 | Schematic of GPI-AP biosynthesis and PGAP1 function. a The synthesis phase of GPI-AP biogenesis in humans and yeast. Substrates and by-products of each reaction are indicated with grey text. Enzymes catalyzing the reactions are indicated with black text with the yeast homologs in brackets. The two steps that may be skipped in humans are indicated with a grey arrow. A question marker denotes unknown participants. **b, c** The remodeling phase of GPI-AP biogenesis in humans (**b**) and yeast (**c**). PGAP1/Bst1, the object of this study, is highlighted in red. **a-c** are redrawn based on Ref. [1] with the recent information from Refs. [2,3]. A question marker denotes unknown information. **d** A model depicting the cross-talk between PGAP1 and the calnexin cycle during the quality control process of GPI-APs. Various components are explained in the figure. The ER membrane is shown as a thick grey line. The simplified schematic is redrawn based on the model proposed in Ref. [4]. **e, f** Schematic of the PGAP1-mediated ERAD pathway (**e**) and RESET or RESET-like pathways (**f**) for the degradation of misfolded GPI-APs. Panel **e** is drawn on conclusions in Ref. [5], and Panel **f** is drawn on models and conclusions from Refs. [6-8]. CANN, calnexin; CMP, cytidine monophosphate; CSP, C-terminal signal peptide; CW, cell wall; DAG, diacylglycerol; DolP, dolichol phosphate; ER, endoplasmic reticulum; ERAD, ER-associated degradation; EtNP, ethanolamine phosphate; FA, fatty acid; GalNAc, acetylgalactosamine; GDP, guanidine phosphate; GlcN, glucosamine; GlcNAc, acetylglucosamine; Glu, glucose; GPI-AP, glycosylphosphatidylinositol-anchored protein; Ino, inositol; MAG, monoacylglycerol; Man, mannose; PE, phosphatidylethonalamine; PM, plasma membrane; RESET, rapid ER (endoplasmic reticulum) stress-induced export; UDP, uridine diphosphate.

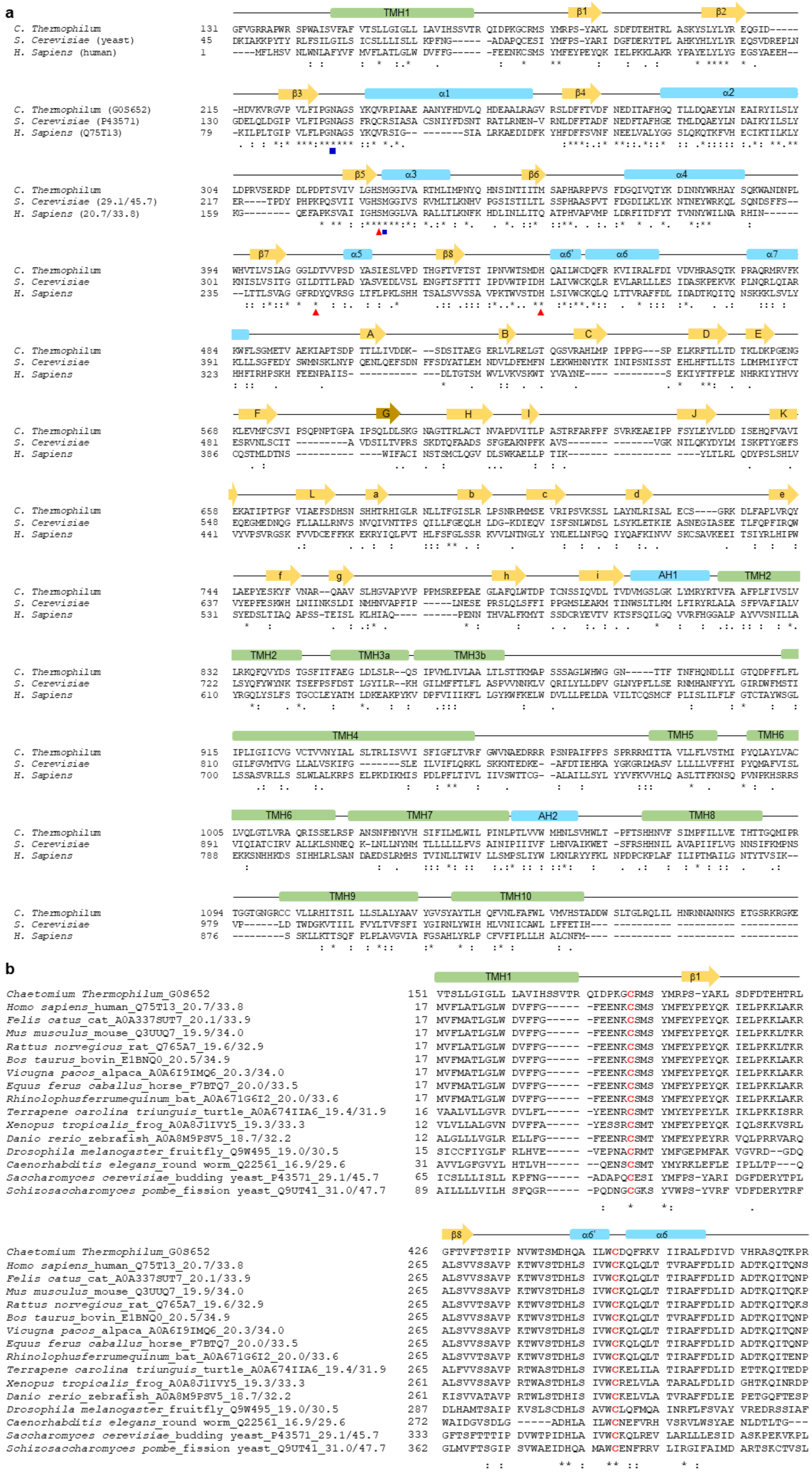

See next page for caption

Fig. S2 | Sequence alignment of PGAP1 orthologs. a Sequence alignment of PGAP1 from *Chaetomium thermophilum*, human, and *Saccharomyces cerevisiae*. Asterisk, colon, and dot indicate identical, conserved, and semi-conserved substitutions, respectively. Secondary structure segments are numbered in accord with Fig. 2a, 2c, 5a, and Fig. S7a. Catalytic triad and oxyanion residues are indicated with a red triangle and a blue square, respectively. The first 130 residues of cPGAP1 and the first 44 residues of yPGAP1, which are predicted to be disordered, are not shown due to their poor sequence homology. **b** Sequence alignment for segments around the conserved cysteine pair (red). Protein sequences were selected from evolutionarily representative species. Uniprot IDs of the sequences and their sequence identity/similarity (%) to cPGAP1 are shown along with the binomial nomenclature and common names. Asterisk, colon, and dot indicate identical, conserved, and semi-conserved substitutions, respectively. Secondary elements were labeled for easier location of the corresponding elements in Fig. S7a. AH, amphipathic helix; TMH, transmembrane helix.

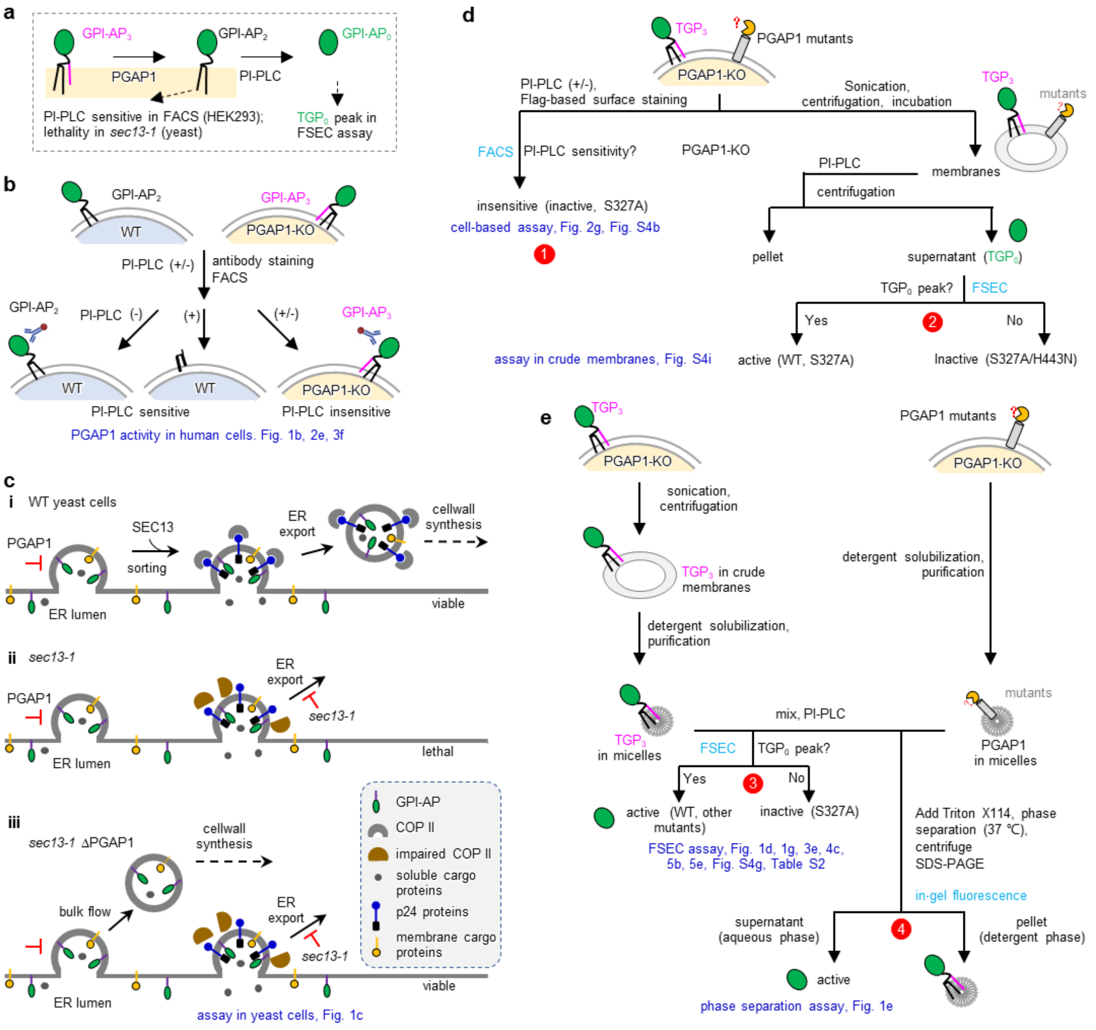

Fig. S3 | Summary of assays. a Principal of the assays. PGAP1 deacylates GPI-AP$_3$ to form GPI-AP$_2$, which can be converted to the water-soluble GPI-AP$_0$. This conversion leads to loss of surface staining in FACS, and delayed elution in FSEC. In yeast, the PGAP1 reaction leads to lethality in *sec13-1* cells. **b** The FACS assay. The surface staining of GPI-AP$_3$ in PGAP1-KO cells is unaffected by PI-PLC treatment unless cells are transfected with functional PGAP1 constructs. **c** Cell-growth assay for PGAP1 function in yeast cells. In wildtype (WT) cells (**i**), PGAP1 inhibits bulk flow vesicles, and GPI-APs are exported by COP vesicles involving Sec13. The *sec13-1* cells (**ii**) fail to assemble COP vesicles at restrictive temperatures and are, therefore, not viable. This lethality is bypassed by the loss of PGAP1 function, as bulk vesicles are no longer suppressed (**iii**). **d** Flowchart of

PGAP1 assays with co-expressed substrate TGP_3. Type **1**: FACS assay; Type **2**: FSEC-based assay for activity in crude membranes. **e** Flowchart of PGAP1 assays using separately expressed and purified TGP_3. In Type **3,** the reaction mix was loaded onto FSEC to separate TGP_3 from TGP_0. In Type **4**, phase separation was performed for the reaction mix using Triton X-114. The detergent and the aqueous phase were analyzed by SDS-PAGE. The TGP bands were visualized by in-gel fluorescence. Figures citing each type of assay are indicated to help with navigation. A question marker denotes PGAP1 mutants with uncertain activity. COP, cytoplasmic coat protein; ER, endoplasmic reticulum; GPI-AP, glycosylphosphatidylinositol-anchored protein; KO, knockout; FACS, fluorescence-activated cell sorting; FSEC, fluorescence-detection size exclusion chromatography; PI-PLC, phosphatidylinositol-specific phospholipase C; TGP, thermostable green fluorescence protein

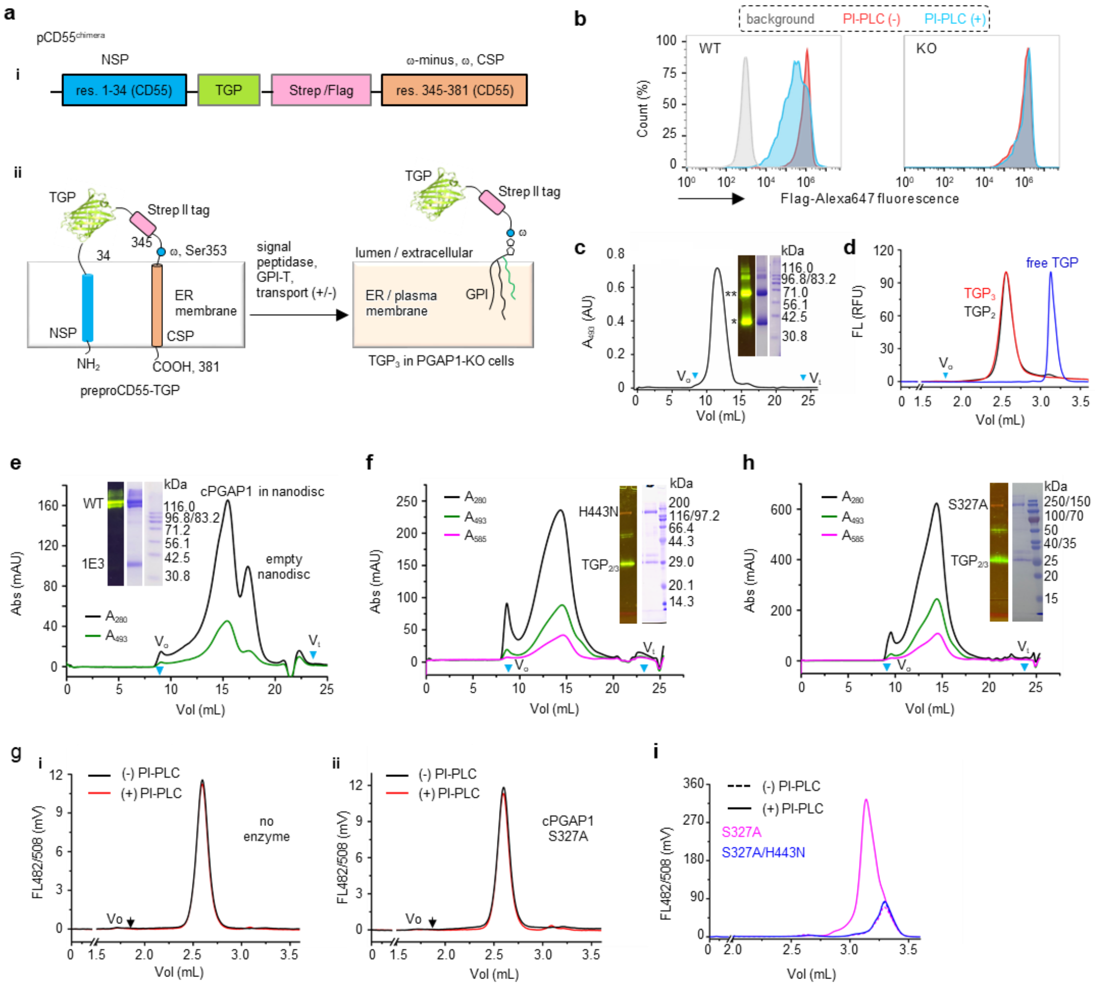

Fig. S4 | Constructs design and purification and reconstitution results. a A simple (**i**) and more realistic (**ii**) cartoon of elements in the chimera GPI-AP (TGP_3). A glycine-serine linker (not shown) flanks the TGP and Strep II tag to avoid possible steric hindrance. +/- (**ii**) indicates compromised vesicle transport of the triacylated GPI-AP due to the absence of PGAP1. **b** FACS of wildtype (WT) HEK293T and hPGAP1-KO cells expressing the chimera protein in **a**, treated with (cyan) or without (orange) PI-PLC. The Flag-tag was used as the marker for staining. The gating strategies are provided in Fig. S12b. **c** Size exclusion chromatography (SEC) and SDS-PAGE (inset) of TGP_3. Absorbance at 493 nm (A_{493}) was monitored. Void (V_o) and total (V_t) volumes of the column are indicated by a triangle. Gels were stained by Coomassie blue (left and middle) or visualized by in-gel fluorescence (right). The gel was visualized on a portable illuminator (TGreen Transilluminator OSE-470, Tiangen Biotech) and the image was taken

using a smartphone. A single and a double asterisk denotes probable monomer and dimer of TGP_3 on SDS-PAGE. The molecular weight of the home-made markers is shown on the left. **d** FSEC of TGP_3 (red), TGP_2 (black), and free TGP (blue). **e** SEC and SDS-PAGE (inset) of cPGAP1 after lipid nanodisc reconstitution. Absorbance at 280 (black) and 493 nm (green) were simultaneously monitored. Contents for each peak were labeled based on SDS-PAGE results. The void (V_o) and total (V_t) volume of the column are indicated by a triangle. SDS-PAGE was visualized by in-gel fluorescence (left) or Coomassie blue staining (middle and right). **f** SEC and SDS-PAGE of cPGAP1^{H443N} in complex with TGP_2. Absorbance at 280 nm (for all proteins, black), 493 nm (for $TGP_{2/3}$, green), and 585 nm (for mCherry-tagged enzyme, magenta) were simultaneously monitored. The gel image was captured using a smartphone with a portable transilluminator. **g** FSEC of the reaction mix from the prolonged assay (see Methods) in the absence (**i**) and presence (**ii**) of cPGAP1 S327A. FSEC profile for the sample treated with (red) and without (black) PI-PLC were recorded. **h** SEC and SDS-PAGE (inset) of cPGAP1^{S327A} in complex with TGP_2. Absorbance at 280 nm (for all proteins, black), 493 nm (for $TGP_{2/3}$, green), and 585 nm (for mCherry-tagged enzyme, magenta) were simultaneously monitored. The gel image was captured using a smartphone with a portable transilluminator. The brightness of the in-gel fluorescence images in **f** and **h** was adjusted as a whole to enhance visibility. Molecular weight of markers are indicated on the side of each gel. **i** FSEC of the supernatant fraction of the crude membranes from hPGAP1-KO cells co-expressing TGP_3 with S327A (magenta) or S327A/H443N (blue) treated with (solid) or without PI-PLC (dash). The 3.1-mL peak corresponds to TGP_0 release from TGP_2 (the product of PGAP1) by PI-PLC. The 3.3-mL peak may correspond to degraded TGP. Results in this figure are from a single experiment. ER, endoplasmic reticulum; FACS, fluorescence-activated cell sorting; FSEC, fluorescence-detection size exclusion chromatography; GPI-AP, glycosylphosphatidylinositol-anchoring protein; KO, knockout; N/CSP, N/C-terminal signal peptide; PI-PLC, phosphatidylinositol-specific phospholipase C; TGP, thermostable green fluorescence protein; ω, the ω-site residue (Ser353 for CD55) where GPI is attached to. The uncropped images of **c**, **e**, **f**, **h** are provided in Fig. S13.

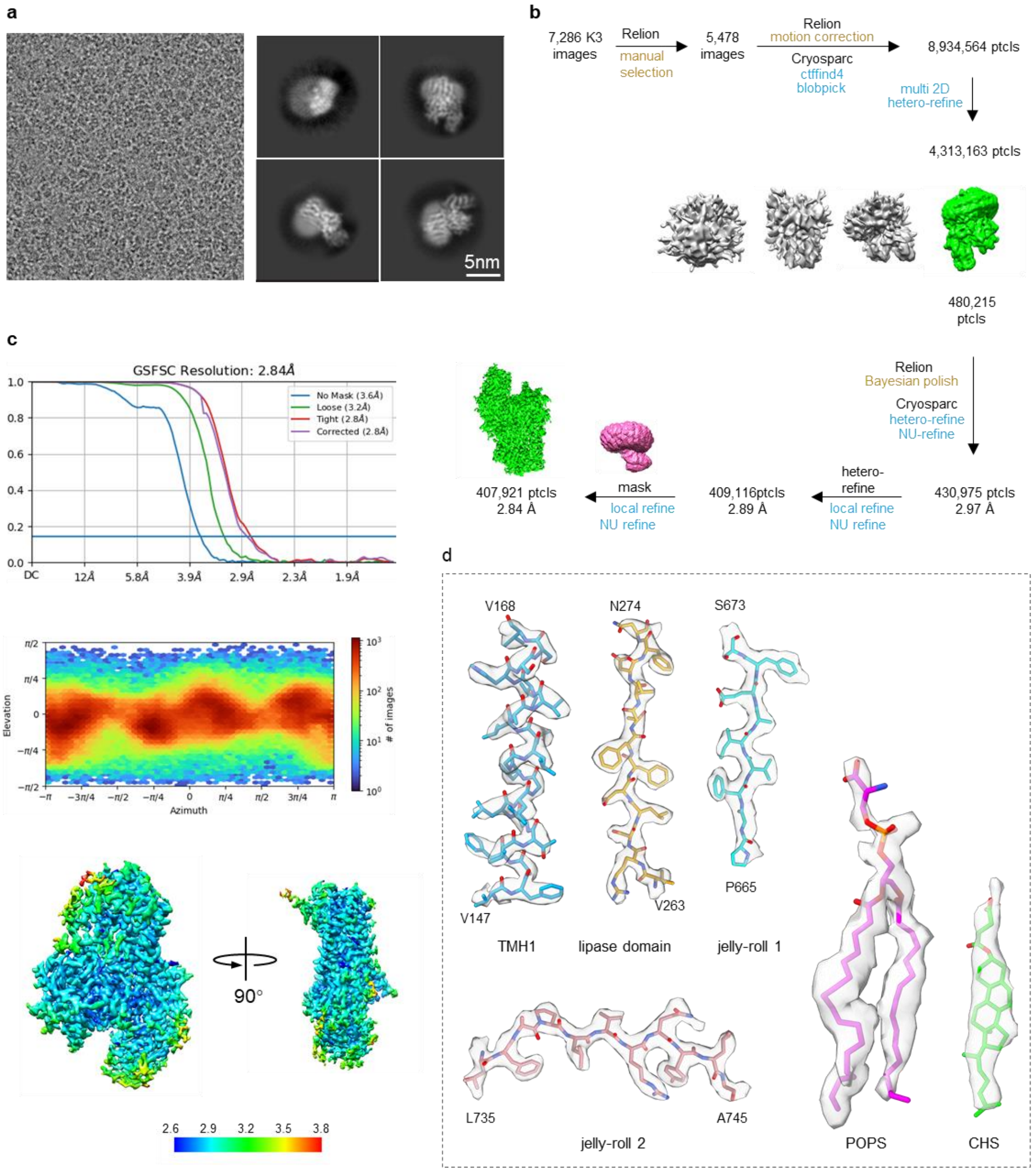

Fig. S5 | Cryo-EM data processing and high-quality density/model fitting exemplary views of the wildtype cPGAP1 in lipid nanodiscs. a Representative cryo-EM micrograph and selected 2D class averages. **b** The workflow of classification and refinement. **c** Top, the nominal resolution of PGAP1 determined by the gold-standard Fourier shell correlation (FSC) curve using the FSC=0.143 criterion; middle, angular distribution heatmap calculated in Cryosparc; bottom, local resolution evaluation. **d** Cryo-EM map density and model of representative protein parts and lipids/detergents. CHS, cholesteryl hemisuccinate; POPS, 1-palmitoyl-2-oleioyl-phosphatidylserine; TMH, transmembrane helix.

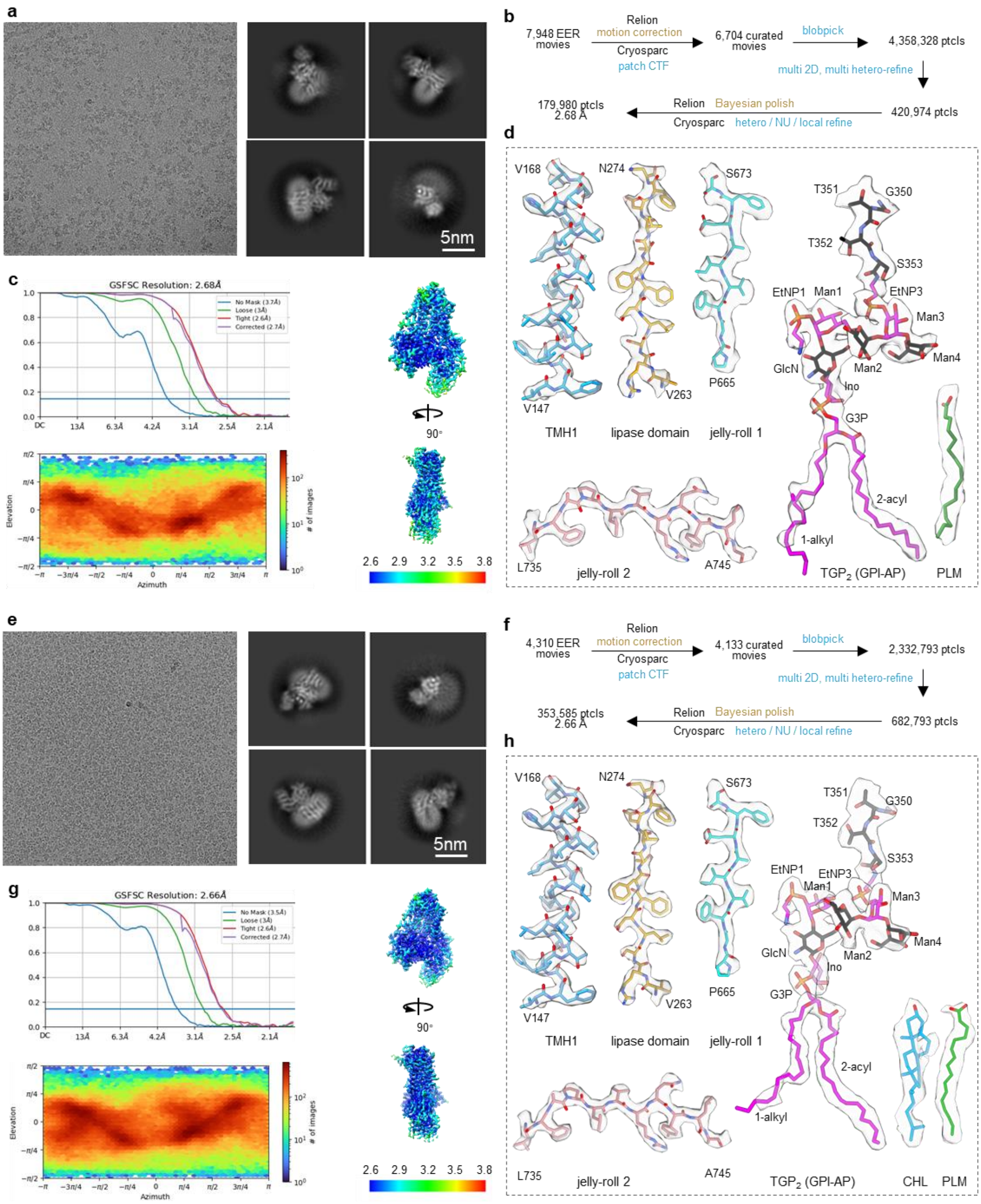

Fig. S6 | Cryo-EM data processing and high-quality density/model fitting exemplary views of liganded cPGAP1 mutants. a Representative cryo-EM micrograph of cPGAP1 H443N in detergents and selected 2D class averages. **b** The workflow of classification and refinement. **c** Top left, the nominal resolution of PGAP1 H443N determined by the gold-standard Fourier shell correlation (FSC) curve using the FSC=0.143 criterion; bottom left, angular distribution heatmap calculated in Cryosparc; right, local resolution evaluation. **d** Cryo-EM map density and model of representative protein parts and lipids/detergents. CHL, cholesterol; PLM, palmitic acid; TMH, transmembrane helix. **e-h** Data processing and density views of cPGAP1 S327A in the same order as **a-d**.

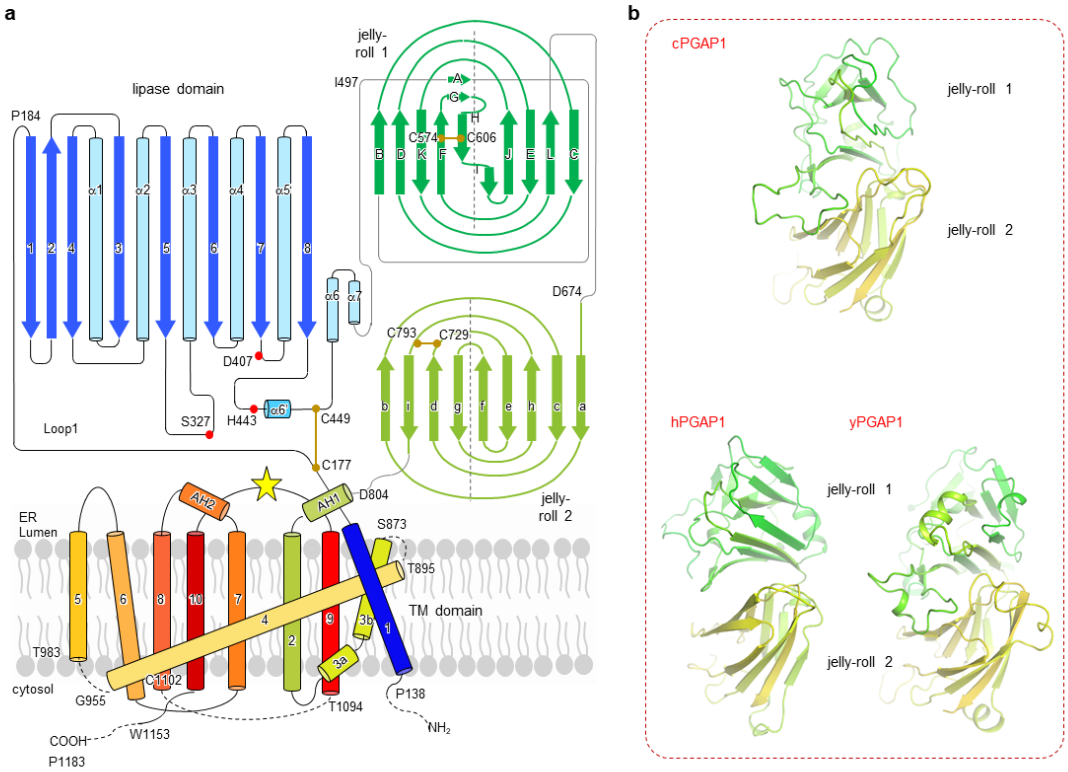

Fig. S7 | Topology and the jelly-roll domains of cPGAP1. a Topology of cPGAP1. Residues at the beginning of each domain are labeled. A star (yellow) near the catalytic triad (red dots) marks the active site where the products are bound. A brown dumbbell indicates disulfide bonds. Dashed lines mark unresolved regions, with the starting and ending residues indicated. AH, amphipathic helix; ER, endoplasmic reticulum; TM, transmembrane. **b** Comparison of the jelly-roll domains between cPGAP1 (top) and the AlphaFold2-predicted[9] hPGAP1 (bottom left) (Uniprot ID Q75T13) and yPGAP1 (bottom right) (Uniprot ID P4357).

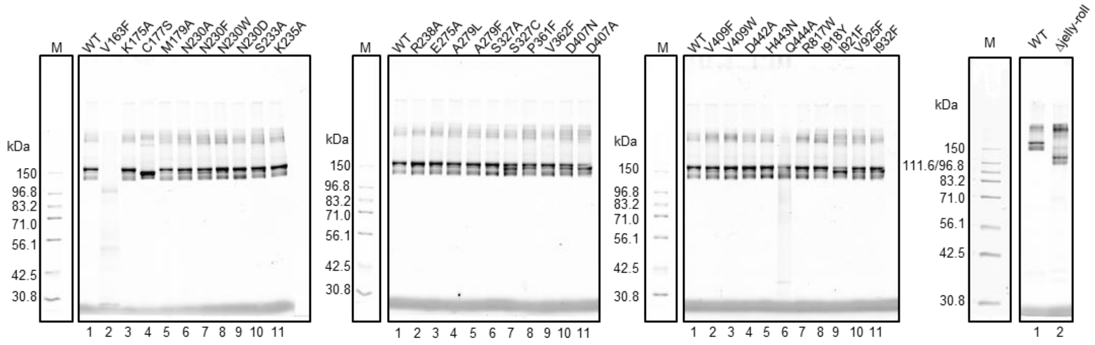

Fig. S8 | Integrity of cPGAP1 mutants on SDS-PAGE. cPGAP1 wildtype (WT) and the indicated mutants were analyzed by SDS-PAGE. Gels were imaged on a Typhoon machine (FLA-9000 controlled by the software Image Reader FLA-9000 Ver.1.0, GE Healthcare) for in-gel fluorescence with the setting for green fluorescence (for the GFP-tagged markers) and for cPGAP1 constructs (mCherry-tagged). The resulting images are merged but the two parts are shown separately to indicate the different fluorescence settings. Molecular weight of the home-made standards[10] are indicated on the left. Results are from a single experiment.

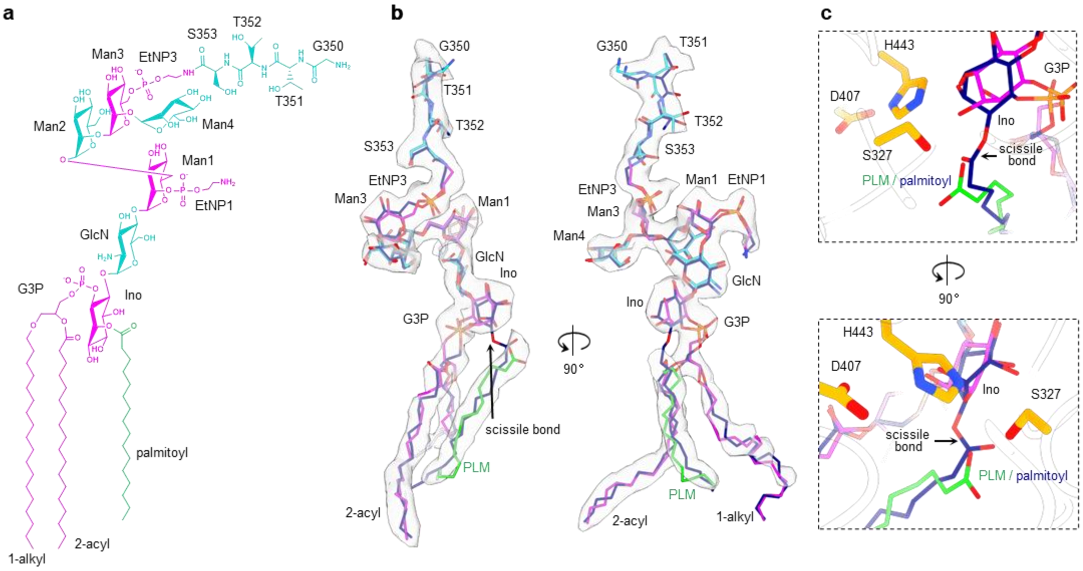

Fig. S9 | Fitting TGP$_3$ into the TGP$_2$/fatty acid density suggests substrate recognition mechanisms. a Chemical structure of TGP$_3$. EtNP2 and TGP are not drawn as they were invisible in the product-bound structures. Various parts are color-coded to match that in Fig. 4a. **b** The fitting of the substrate TGP$_3$ (blue) into the density of products in cPGAP1^{H443N}. TGP$_2$ (product 1) is colored alternatingly with magenta and cyan, while palmitic acid (PLM, product 2) is colored green. **c** Expanded view of the catalytic site. The catalytic triad (orange), TGP$_3$ (blue), TGP$_2$ (magenta), and PLM (green) are shown as stick representations. EtNP, ethanolamine phosphate; G3P, glycerol 3-phosphate; GlcN, glucosamine; Ino, inositol; Man, mannose; PLM, palmitic acid; TGP, thermostable green fluorescence protein.

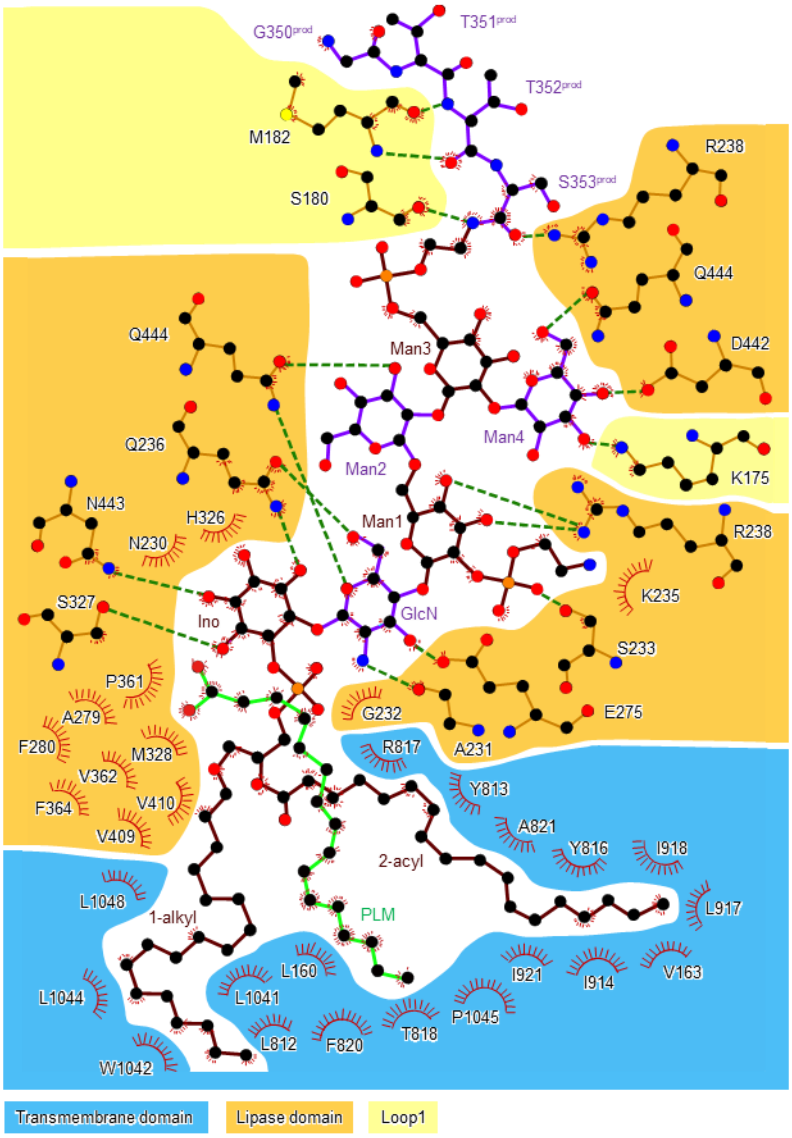

Fig. S10 | Simplified LigPlot[11] view of the product-cPGAP1 interactions. Hydrophobic interactions are indicated by eye slashes and H-bonds are indicated by dashed lines. The palmitic acid (PLM) is colored green. The GPI moiety is colored alternatively and protein subunits are shaded by the color scheme used in Fig. 2a, 2b, and Fig. 4. EtNP, ethanolamine phosphate; GlcN, glucosamine; GPI, glycosylphosphatidylinositol; Ino, inositol; Man, mannose; PLM, palmitic acid.

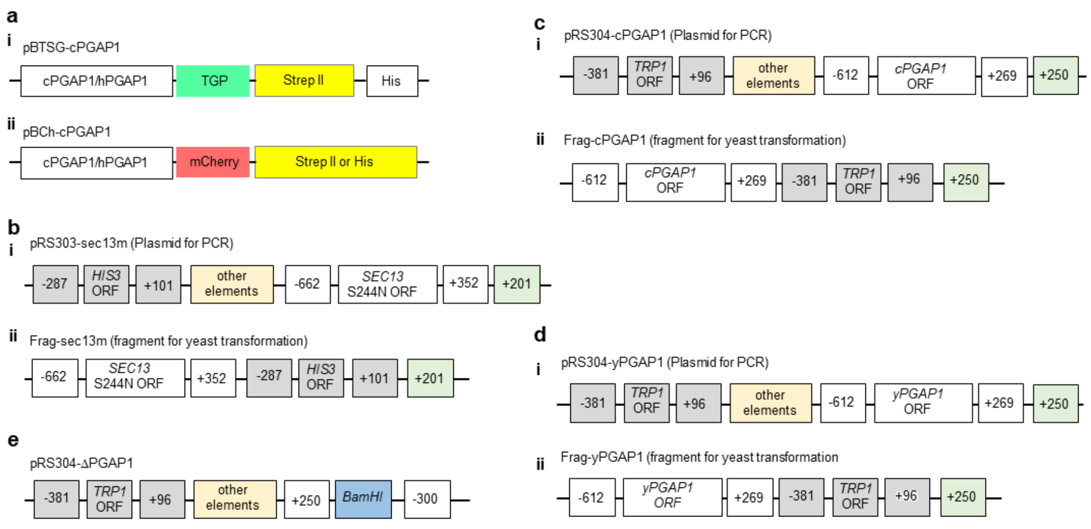

Fig. S11 | Simplified schematics of constructs used in this study. a The construct that expressses cPGAP1 for the structural determination of cPGAP1^{apo} (**i**), and for the assays of all cPGAP1 variants (Strep-tag) and the structural study of cPGAP1 H443N and S327A (His-tag) (**ii**). **b-d** The construct (**i**) and the fragment (**ii**) used for the generation of the *sec13-1* mutant (**b**), the replacement of yPGAP1 with cPGAP1 (**c**) or yPGAP1 itself (**d**). **e** The construct used for the generation of yPGAP1-KO cells. Positive and negative numbers indicate nucleotide positions downstream (3’-direction) and upstream (5’-direction) of open reading frame (ORF) of indicated genes, respectively. *HIS* and *TRP* are genes encoding the nutrient markers for selection of positive integration. In **b-d**, fragments (**ii**) were obtained by Gibson assembly of several fragments amplified by polymerase chain reaction (PCR) using the constructs in (**i**) as the template (See Methods). KO, knockout; TGP, thermostable fluorescence protein.

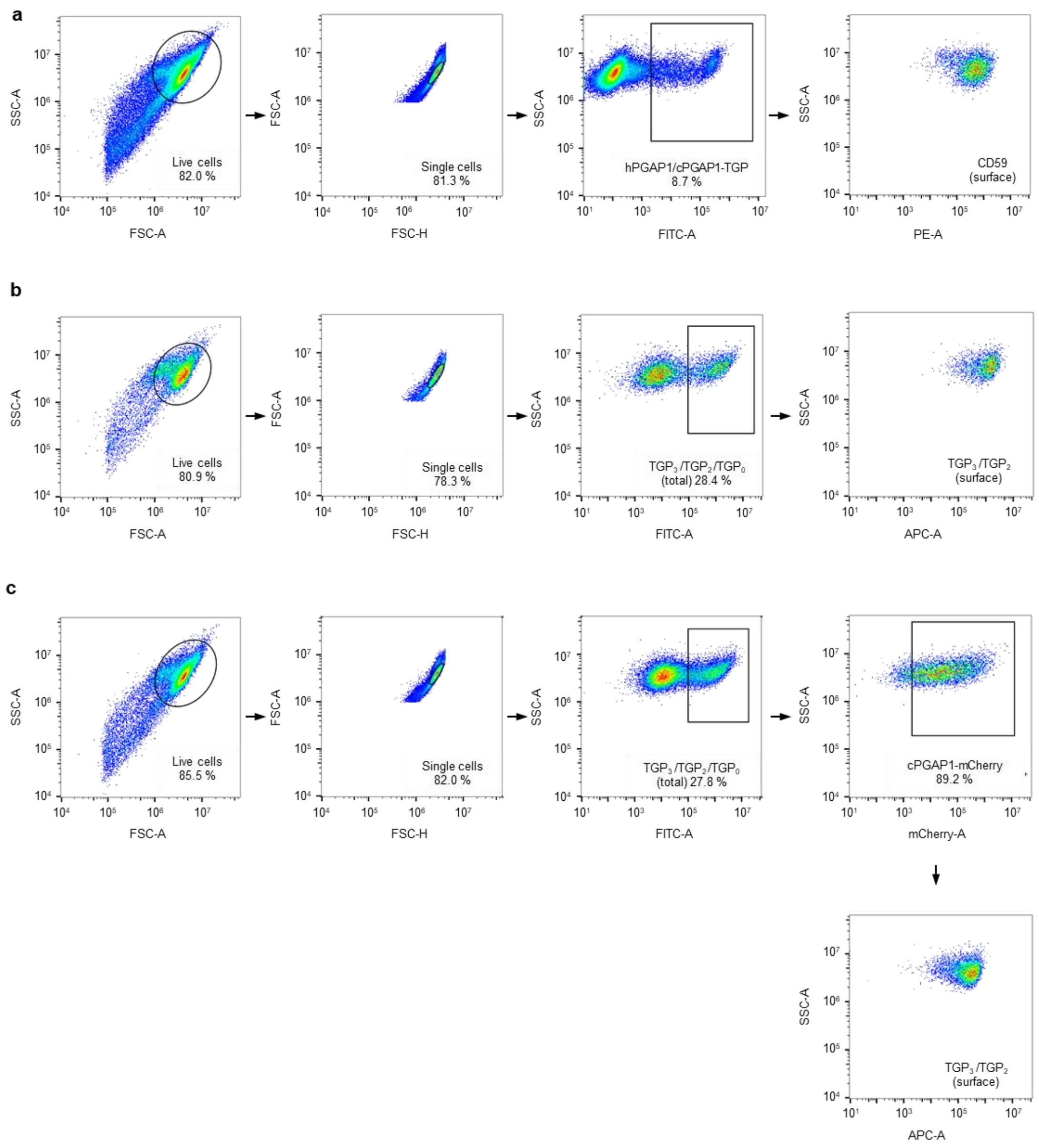

Fig. S12 | Gating strategies for fluorescence-activated cell sorting. a The cells were first gated to select living cells and single cells. The expression of TGP-fused hPGAP1/cPGAP1/mutants was gated by the fluorescence of TGP (488/525 nm). This population was further analyzed for PE-positivity (561/585 nm) as an indication of the cell surface staining of CD59 via its antibody (Fig. 1b, 2e, 3f). **b** Cells were first gated to select living cells and single cells. Cells expressing Flag-tagged $TGP_3/TGP_2/TGP_0$ were gated by TGP fluorescence to eliminate non-expressing cells (total TGP expression). The TGP-positive cells were further analyzed by APC fluorescence (638/660 nm from anti-Flag antibodies), which indicates cell surface expression of TGP_3/TGP_2 (Fig. S4b). **c** Cells were first gated

to select living cells and single cells. Cells expressing Flag-tagged TGP_3/TGP_2/TGP_0 were gated by TGP fluorescence to eliminate non-expressing cells (total TGP expression). The TGP-positive cells were further gated by mCherry fluorescence to select cells expressing cPGAP1/mutants (mCherry fusion). Finally, APC fluorescence (from anti-Flag antibodies) was used to gate cells displaying GPI-anchored TGP_3/TGP_2 on the cell surface (Fig. 2g). The data distribution is shown as a heat map with low counts in blue and high counts in red. APC, allophycocyanin; FSC A/H, forward scatter area/height; FITC, fluorescein isothiocyanate (channel used in FACS analysis which detects TGP); GPI, glycosylphosphatidylinositol; PE, phycoerythrin (channel used for CD59 staining); SSC A, side scatter area; TGP, thermostable green fluorescence protein.

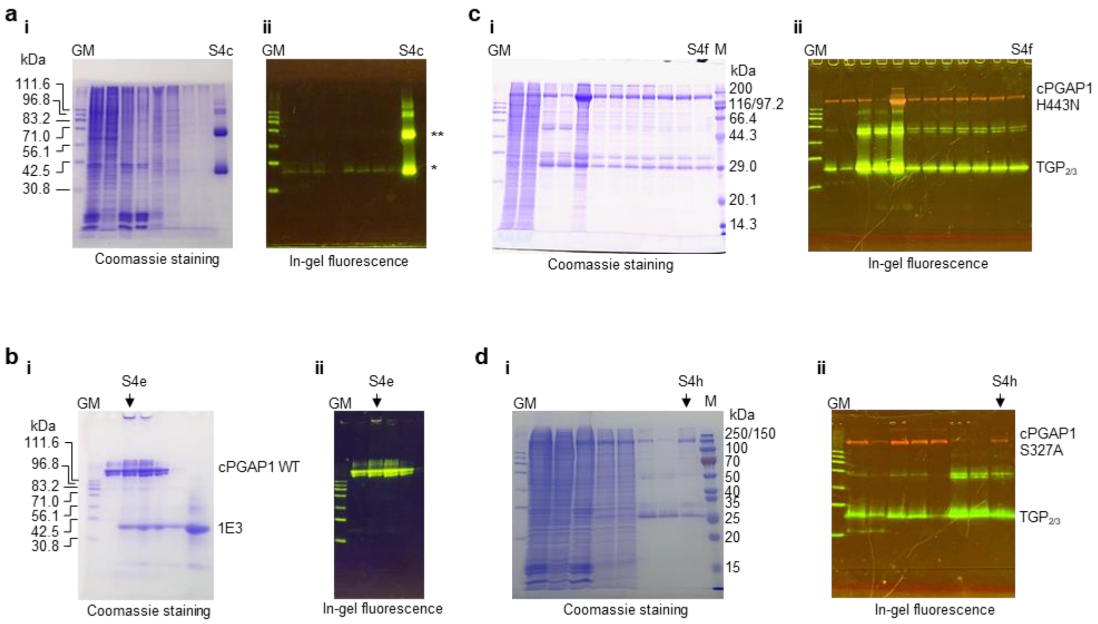

Fig. S13 | Uncropped images for Fig. S4. a Fig. S4c. A single and a double asterisk denotes probable monomer and dimer of TGP_3. **b** Fig. S4e. **c** Fig. S4f. **d** Fig. S4h. Both the Coomassie Blue images (**i**) and in-gel fluorescence images (**ii**) of each gel are shown. Lanes are labelled only for those shown in Fig. S4. Molecular weights of the home-made GFP markers (GM) and standard markers (M) are indicated on the side (left, GFP markers; right, standard markers). In-gel fluorescence images were taken with a portable transilluminator. The Coomassie-stained gel images were captured using a smartphone.

Table S1 | Cryo-EM data collection and refinement statistics.

	cPGAP1^{apo}	cPGAP1^{S327A}	cPGAP1^{H443N}
**Data collection and processing**			
Magnification	105,000	130,000	130,000
Voltage (kV)	300	300	300
Electron exposure (e^{-}/Å^2)	52	50	50
Defocus range (μm)	-1.2 to -2.5	-1.5 to -2.0	-1.5 to -2.0
Pixel size (Å)	0.832	0.932	0.932
Symmetry imposed	C1	C1	C1
Initial particle images (no.)	4,313,163	2,332,793	4,385,328
Final particle images (no.)	407,921	353,585	179,980
Map resolution (Å)	2.84	2.66	2.68
FSC threshold	0.143	0.143	0.143
Map resolution range (Å)	2.6-3.8	2.6-3.8	2.6-3.8
**Refinement**			
Model resolution (Å)	3.1	2.9	2.9
FSC threshold	0.5	0.5	0.5
Map sharpening *B* factor (Å^2)	134.6	84.3	103.3
**Model composition**			
Non-hydrogen atoms	7,549	7,748	7,684
Protein residues	927	961	958
Ligands	5	4	3
B factor (Å^2)			
Protein	40.99	67.17	72.78
Ligand	46.70	65.79	62.96
R.m.s. deviations			
Bond lengths (Å)	0.015	0.004	0.014
Bond angles (°)	1.910	0.624	0.865
Validation			
MolProbity score	2.27	1.46	1.57
Clashscore	13.25	6.20	6.10
Ramachandran plot			
Favored (%)	98.03	97.37	97.26
Allowed (%)	1.97	2.63	2.74
Outliers (%)	0.00	0.00	0.00

Table S2. Activity of cPGAP1 mutants from the fluorescence-detection size exclusion chromatography assay using the separately expressed and purified substrate TGP_3.

Construct	Activity, %	s.e.m.[a]	Yield[b]
WT	100.0	10.0	++++
K175A	146.29	14.36	+++
C177S	6.9	0.26	+
M179A	178.71	16.27	++
N230A	66.21	3.23	+
N230F	36.93	0.75	+
N230W	30.57	1.95	++++
N230D	94.56	3.32	++
S233A	92.90	2.90	++
K235A	204.64	6.82	+++
R238A	182.54	9.91	++
E275A	192.24	6.92	+++
A279L	67.64	2.60	+
A279F	31.34	3.99	++
S327A	0.03	0.01	++++
S327C	7.44	0.23	++++
P361F	20.19	0.44	+
V362F	141.88	4.04	+
D407N	21.86	0.27	++
D407A	8.40	0.80	+++
V409F	51.86	3.12	++
V409W	33.36	3.10	+++
D442A	188.07	6.83	++
H443N	0.38	0.00	++++
Q444A	176.59	4.85	+
R817W	68.69	9.15	++
I918Y	28.93	3.19	++
I921F	37.14	2.63	++
V925F	107.11	6.94	+++
I932F	118.73	2.49	++

[a]Standard error of the mean from three independent experiments. [b]Purification yield (milligrams of protein per deciliter of culture). ++++, >1; +++, 0.5-1; ++, 0.2-0.5; and +, < 0.2.

Supplementary References

1. Kinoshita, T. Biosynthesis and biology of mammalian GPI-anchored proteins. *Open Biol* **10**, 190290 (2020).
2. Wang, Y. et al. Genome-wide CRISPR screen reveals CLPTM1L as a lipid scramblase required for efficient glycosylphosphatidylinositol biosynthesis. *Proc Natl Acad Sci USA* **119**, e2115083119 (2022).
3. Cao, S.-Y., Liu, Y.-S., Gao, X.-D., Kinoshita, T. & Fujita, M. A lipid scramblase TMEM41B is involved in the processing and transport of GPI-anchored proteins. *J Biochem* **174**, 109-123 (2023).
4. Guo, X.Y., Liu, Y.S., Gao, X.D., Kinoshita, T. & Fujita, M. Calnexin mediates the maturation of GPI-anchors through ER retention. *J Biol Chem* **295**, 16393-16410 (2020).
5. Fujita, M., Yoko, O.T. & Jigami, Y. Inositol deacylation by Bst1p is required for the quality control of glycosylphosphatidylinositol-anchored proteins. *Mol Biol Cell* **17**, 834-50 (2006).
6. Satpute-Krishnan, P. et al. ER stress-induced clearance of misfolded GPI-anchored proteins via the secretory pathway. *Cell* **158**, 522-33 (2014).
7. Sikorska, N. et al. Limited ER quality control for GPI-anchored proteins. *J Cell Biol* **213**, 693-704 (2016).
8. Lemus, L. et al. Post-ER degradation of misfolded GPI-anchored proteins is linked with microautophagy. *Curr Biol* **31**, 4025-4037.e5 (2021).
9. Jumper, J. et al. Highly accurate protein structure prediction with AlphaFold. *Nature* **596**, 583-589 (2021).
10. Cai, H., Yao, H., Li, T., Tang, Y. & Li, D. High-level heterologous expression of the human transmembrane sterol Δ8,Δ7-isomerase in Pichia pastoris. *Protein Expr Purif* **164**, 105463 (2019).
11. Laskowski, R.A. & Swindells, M.B. LigPlot+: multiple ligand-protein interaction diagrams for drug discovery. *J Chem Inf Model* **51**, 2778-86 (2011).
