# Supplementary material for: Molecular basis of the inositol deacylase PGAP1 involved in quality control of GPI-AP biogenesis
Source: Nat Commun. 2024 Jan 2;15:8. doi: 10.1038/s41467-023-44568-2 (PMC10761859; doi:10.1038/s41467-023-44568-2)
Supplement: Supplementary file 3 — Reporting Summary [file 41467_2023_44568_MOESM3_ESM.pdf]

nature portfolio

Corresponding author(s): Dianfan Li, Qianhui Qu

Last updated by author(s): Dec 13, 2023

Reporting Summary

Nature Portfolio wishes to improve the reproducibility of the work that we publish. This form provides structure for consistency and transparency in reporting. For further information on Nature Portfolio policies, see our Editorial Policies and the Editorial Policy Checklist.

Statistics

For all statistical analyses, confirm that the following items are present in the figure legend, table legend, main text, or Methods section.

n/a	Confirmed	
☐	☒	The exact sample size (*n*) for each experimental group/condition, given as a discrete number and unit of measurement
☐	☒	A statement on whether measurements were taken from distinct samples or whether the same sample was measured repeatedly
☐	☒	The statistical test(s) used AND whether they are one- or two-sided<br>*Only common tests should be described solely by name; describe more complex techniques in the Methods section.*
☒	☐	A description of all covariates tested
☐	☒	A description of any assumptions or corrections, such as tests of normality and adjustment for multiple comparisons
☐	☒	A full description of the statistical parameters including central tendency (e.g. means) or other basic estimates (e.g. regression coefficient) AND variation (e.g. standard deviation) or associated estimates of uncertainty (e.g. confidence intervals)
☐	☒	For null hypothesis testing, the test statistic (e.g. *F*, *t*, *r*) with confidence intervals, effect sizes, degrees of freedom and *P* value noted<br>*Give P values as exact values whenever suitable.*
☒	☐	For Bayesian analysis, information on the choice of priors and Markov chain Monte Carlo settings
☒	☐	For hierarchical and complex designs, identification of the appropriate level for tests and full reporting of outcomes
☒	☐	Estimates of effect sizes (e.g. Cohen's *d*, Pearson's *r*), indicating how they were calculated

Our web collection on statistics for biologists contains articles on many of the points above.

Software and code

Policy information about availability of computer code

Data collection	Preparative size exclusion chromatography, ChromLab 5.0.2.11; Fluorescence-detection size-exclusion chromatography, LabSolutions 5.87; Optical density of yeast growth, SoftMax Pro 7.1.2; Cryo-EM data collection, Serial EM 3.8.0 for cPGAP1 apo, EPU for cPGAP1 H443N and cPGAP1 S327A; In-gel fluorescence, TGreen Transilluminator OSE-470 (Fig. S4c, e, f, h), In-gel fluorescence, FLA-9000, Image Reader FLA-9000 Ver.1.0 (Fig. 1e, Fig. S8); Flow cytometry, Beckman CytoFlex LX, CytExpert 2.4.0.28; Cell sorting, BD FACS Diva 8.0.3.
Data analysis	Cryo-EM data processing, Relion 3.1, CTFFIND4, and CryoSPARC 4.2.0; 3D-model building, Coot 0.9.6; Structure refinement, Phenix 1.19.2-4158; Structure visualization, PyMOL 2.3.3 and ChimeraX1.1; FACS data analysis, FlowJo v10.8.1; Activity analyzation, GrapPad Prism 8; Fluorescence-detection size-exclusion chromatography data analyzation and visualization, LabSolutions 5.87.

For manuscripts utilizing custom algorithms or software that are central to the research but not yet described in published literature, software must be made available to editors and reviewers. We strongly encourage code deposition in a community repository (e.g. GitHub). See the Nature Portfolio guidelines for submitting code & software for further information.

Data

Policy information about availability of data

All manuscripts must include a data availability statement. This statement should provide the following information, where applicable:

- Accession codes, unique identifiers, or web links for publicly available datasets
- A description of any restrictions on data availability
- For clinical datasets or third party data, please ensure that the statement adheres to our policy

The coordinates for the model generated in this study have been deposited in the PDB under accession code 8K9Q [https://doi.org/10.2210/pdb8K9Q/pdb] (cPGAP1apo), 8K9T [https://doi.org/10.2210/pdb8K9T/pdb] (cPGAP1S327A), and 8K9R [https://doi.org/10.2210/pdb8K9R/pdb] (cPGAP1H443N). The cryo-EM density maps generated in this study have been deposited in the Electron Microscopy Data Bank (EMDB) under accession code EMD-36995 [https://www.ebi.ac.uk/pdbe/entry/emdb/EMD-36995] (cPGAP1apo), EMD-36997 [https://www.ebi.ac.uk/pdbe/entry/emdb/EMD-36997] (cPGAP1S327A), and EMD-36996 [https://www.ebi.ac.uk/pdbe/entry/emdb/EMD-36996] (cPGAP1H443N). Uncropped images of Fig. 1e and tabular data for Figs. 1c, 1g, 3e, 4c, 5b and 5e are provided in the Source Data file. The uncropped images of Figs. S4c, S4e, S4f and S4h are provided in Fig. S13. Source data are provided in this paper.

Research involving human participants, their data, or biological material

Policy information about studies with human participants or human data. See also policy information about sex, gender (identity/presentation), and sexual orientation and race, ethnicity and racism.

Reporting on sex and gender	n/a
Reporting on race, ethnicity, or other socially relevant groupings	n/a
Population characteristics	n/a
Recruitment	n/a
Ethics oversight	n/a

Note that full information on the approval of the study protocol must also be provided in the manuscript.

Field-specific reporting

Please select the one below that is the best fit for your research. If you are not sure, read the appropriate sections before making your selection.

☒ Life sciences ☐ Behavioural & social sciences ☐ Ecological, evolutionary & environmental sciences

For a reference copy of the document with all sections, see nature.com/documents/nr-reporting-summary-flat.pdf

Life sciences study design

All studies must disclose on these points even when the disclosure is negative.

Sample size	The sample size (n=3) is stated in the figure legends. Sample size was chosen based on previous experience and similar reports in the literature (Nat Commun 2022 13:2617). No statistical methods were used to predetermine sample size.
Data exclusions	No data were excluded from the analysis.
Replication	All experimental results in this study are either from three independent experiments, or presented as a typical of at least three experiments unless specially stated. Similar results were obtained in repeated experiments using different cell batches and different plasmid preps and attempts to repeat the experiments were successful. Use of statistical methods have been described in relevant figure legends.
Randomization	The FACS assays sample a large number of cells that are from a single colony. Randomization was not relevant for this study. The yeast growth assay was performed with three single colonies (picked randomly). Biochemical and biophysical experiments, including protein purification, SDS-PAGE, TLC, FSEC assay, phase separation, and cryo-EM data collection, are not subjective and hence do not require randomization.
Blinding	Because the data collection and analysis procedures were not subjective, there was no need for blinding.

Reporting for specific materials, systems and methods

We require information from authors about some types of materials, experimental systems and methods used in many studies. Here, indicate whether each material, system or method listed is relevant to your study. If you are not sure if a list item applies to your research, read the appropriate section before selecting a response.

Materials & experimental systems

n/a	Involved in the study
☐	☒ Antibodies
☐	☒ Eukaryotic cell lines
☒	☐ Palaeontology and archaeology
☒	☐ Animals and other organisms
☒	☐ Clinical data
☒	☐ Dual use research of concern
☒	☐ Plants

Methods

n/a	Involved in the study
☒	☐ ChIP-seq
☐	☒ Flow cytometry
☒	☐ MRI-based neuroimaging

Antibodies

Antibodies used	Phycoerythrin (PE)-labeled CD59 antibody (12-0596-42, clone OV9A2, Thermo Fisher Scientific, 1 : 500 dilution); Alexa Fluor 647 conjugated Flag antibody (D6W5B) Rabbit mAb (Cat.15009S, Cell Signaling Technology, 1:100 dilution)
Validation	The validation of the Phycoerythrin (PE)-labeled CD59 antibody is conducted by the manufacturer. This OV9A2 monoclonal antibody reacts with human CD59, which is validated by staining the normal human peripheral blood cells with Mouse IgG1 K Isotype Control PE (Product # 12-4714-81) as the control and then analyzing with FACS. The information can be found with the link: https://www.thermofisher.cn/order/genome-database/dataSheetPdf?producttype=antibody&productsubtype=antibody_primary&productId=12-0596-42&version=216.<br><br>The validation of the Alexa Fluor 647 conjugated Flag antibody (D6W5B) is conducted by the manufacturer. This D6W5B antibody is conjugated to Alexa Fluor® 647 fluorescent dye and tested in-house for direct flow cytometry analysis in monkey cells. This antibody is expected to exhibit the same species cross-reactivity as the unconjugated DYKDDDDK Tag (D6W5B) Rabbit mAb (Binds to same epitope as Sigma's Anti-FLAG® M2 Antibody) #14793. DYKDDDDK Tag (D6W5B) Rabbit mAb (Binds to same epitope as Sigma's Anti-FLAG® M2 Antibody) (Alexa Fluor® 647 Conjugate) detects exogenously expressed DYKDDDDK proteins in cells. The antibody recognizes the DYKDDDDK peptide, which is the same epitope recognized by Sigma's Anti-FLAG® antibodies, fused to either the amino-terminus or carboxy-terminus of the target protein. The information can be found with the link: https://www.cellsignal.com/products/antibody-conjugates/dykddddk-tag-d6w5b-rabbit-mab-binds-to-same-epitope-as-sigma-s-anti-flag-m2-antibody-alexa-fluor-647-conjugate/15009.

Eukaryotic cell lines

Policy information about cell lines and Sex and Gender in Research

Cell line source(s)	HEK293T cells (Cat. CRL-3216, ATCC); Expi293 cells (Cat. A14527, ThermoFisher Scientific); PIGK knock-out cell line, generated in our previous study (Nat Commun 2022 13:2617); PGAP1 knock-out cell line, generated in this study.
Authentication	HEK-293 cells and Expi293 cells were not authenticated. The knock-out cell lines were verified using PCR, sequencing, and FACS analysis. Cells were maintained at lowest passage numbers possible to maintain identity.
Mycoplasma contamination	The cell lines were not tested for mycoplasma contamination.
Commonly misidentified lines<br>(See ICLAC register)	No commonly misidentified cell lines were used in this study.

Plants

Seed stocks	*Report on the source of all seed stocks or other plant material used. If applicable, state the seed stock centre and catalogue number. If plant specimens were collected from the field, describe the collection location, date and sampling procedures.*
Novel plant genotypes	*Describe the methods by which all novel plant genotypes were produced. This includes those generated by transgenic approaches, gene editing, chemical/radiation-based mutagenesis and hybridization. For transgenic lines, describe the transformation method, the number of independent lines analyzed and the generation upon which experiments were performed. For gene-edited lines, describe the editor used, the endogenous sequence targeted for editing, the targeting guide RNA sequence (if applicable) and how the editor was applied.*
Authentication	*Describe any authentication procedures for each seed stock used or novel genotype generated. Describe any experiments used to assess the effect of a mutation and, where applicable, how potential secondary effects (e.g. second site T-DNA insertions, mosiacism, off-target gene editing) were examined.*

Flow Cytometry

Plots

Confirm that:

☒ The axis labels state the marker and fluorochrome used (e.g. CD4-FITC).

☒ The axis scales are clearly visible. Include numbers along axes only for bottom left plot of group (a 'group' is an analysis of identical markers).

☒ All plots are contour plots with outliers or pseudocolor plots.

☒ A numerical value for number of cells or percentage (with statistics) is provided.

Methodology

Sample preparation	Transfected wide type and human PGAP1-KO HEK293T cells were washed with PBS and collected by trypsin digestion. Cells were washed once again before treated with or without 10 µg PI-PLC at 37 ºC for 1.5 h. Cells were washed once after the PI-PLC treatment and followed by antibody staining.<br>For cell surface CD59 staining, phycoerythrin (PE)-labeled anti-CD59 antibody (Cat. 12-0596-42, Thermo Fisher Scientific) was used as a 500-fold dilution for incubation with the cells for 15 min at dark. Cells were washed once and resuspended in 0.3 mL of PBS before analyzed by flow cytometry (Beckman CytoFlex LX) using two wavelength-pairs (488/525 nm for TGP-tagged PGAP1, 561/585 nm for PE).<br>For cell surface Flag-tagged TGP3/TGP2 staining, Alexa647-labeled anti-Flag antibody (Cat.15009S, Cell Signaling Technology) was used at a 100-fold dilution for 30 min-incubation at room temperature prevented from light. Cells were treated as above and analyzed by flow cytometry (Beckman CytoFlex LX) using three wavelength pairs (488/525 nm for the expression of the TGP-tagged substrate, 561/610 nm for mCherry-tagged PGAP1, and 638/660 nm for surface display of TGP2 / TGP3).
Instrument	Beckman CytoFlex LX
Software	CytExpert 2.4.0.28 was used to collect the flow cytometry data. FlowJo v10.8.1 was used to analyze the flow cytometry data.
Cell population abundance	The commercial HEK cell lines has a cell population of 100%. The knockout cell lines we generated in this study was developed from single colonies and also have a cell population of 100%. Typically 40,000 cells were analyzed for each sample.
Gating strategy	In all FACS runs, cells were first gated to select living cells and single cells.<br>For cell surface staining of CD59, the expression of TGP-fused hPGAP1/cPGAP1/mutants was gated by the fluorescence of TGP (488/525 nm). This population was further analyzed for phycoerythrin (PE)-positivity (561/585 nm) as an indication of the cell surface staining of CD59 via its antibody (Fig. 1b, 2e, 3f).<br>For cell surface staining of Flag-tagged TGP3/TGP2, cells expressing Flag-tagged TGP3/TGP2/TGP0 were first gated by TGP fluorescence to eliminate non-expressing cells (total TGP expression). The TGP-positive cells were further analyzed by allophycocyanin (APC) fluorescence (638/660 nm from anti-Flag antibodies), which indicates cell surface expression of Flag-tagged TGP3/TGP2 (Fig. S4b). In cells co-expressing mCherry-tagged cPGAP1/mutants, the TGP positive cells were gated by mCherry fluorescence (for cPGAP1 expression) before being gated by APC fluorescence (from anti-Flag antibodies) for the surface expression of TGP3/TGP2 (Fig. 2g).

☒ Tick this box to confirm that a figure exemplifying the gating strategy is provided in the Supplementary Information.
